# Evaluation Method of Physical Education Teaching and Training Quality Based on Deep Learning

**DOI:** 10.1155/2022/1680888

**Published:** 2022-06-23

**Authors:** Hong Wang, Fei Yang, Xingyang Xing

**Affiliations:** ^1^Department of Sport and Healthcare, Namseoul University, Cheonan, Republic of Korea; ^2^Department of Sports Art, Hebei Institute of Physical Education, Hebei, Shijiazhuang, China; ^3^Department of Physical Education, Hebei Institute of Physical Education, Hebei, Shijiazhuang, China

## Abstract

To solve the problems of great difficulty and low accuracy in the evaluation of physical education teaching results, an evaluation method of physical education teaching and training quality based on deep learning is proposed. The evaluation index system is constructed based on the teaching content, teaching attitude, teaching content, teaching method, and teaching effects that affect the teaching quality. After the influence factors of each index are quantified, the resolution coefficient of the index is dynamically taken, the index correlation relationship based on weight is established, and the score distribution and score progress are taken as the evaluation results. The test results show that the correlation coefficient between the evaluation result of the design method and the actual results is 0.9430, and the evaluation accuracy is 94.73%.

## 1. Introduction

The implementation of physical education is mainly done through physical education teaching activities. The Ministry of Education attaches great importance to teaching reform. The new curriculum standard in December 2011 emphasizes that curriculum standards should be the main basis for teaching in the process of further promoting teaching reform. All localities should actively guide teachers to organize teaching activities in strict accordance with curriculum standards, reasonably grasp teaching capacity and teaching difficulty, actively adjust teaching concepts and teaching behavior, pay full attention to the cultivation and improvement of students' learning initiative and enthusiasm, and strive to control their schoolwork burden [1–3]. In this context, in-depth research on physical education teaching is really necessary. In recent years, the decline in young students' physique has become an indisputable fact. Although the physical decline of young students is not caused by school physical education unilaterally, there is no doubt that if school physical education is done well, it can enhance students' physical fitness to a great extent, and the traditional physical education teaching mode that only pays attention to knowledge teaching and skill training is obviously incompatible with this [4, 5]. As far as physical education is concerned, only when students master how to study independently in a physical education classroom, learn how to adapt and adjust themselves, and then develop into a way of lifelong physical education, physical education can be regarded as a real success [6–8]. However, the reality is not very optimistic. The current imperfect evaluation model of physical education teaching makes most students and parents only care about physical examination results and ignore lifelong physical education. There are many reasons for this situation, and the current backward concept of physical education teaching evaluation is undoubtedly the main reason for this phenomenon. Under the background of the rise of national fitness as a national strategy, there has been a general decline and disharmony in the physique of primary and middle school students. The responsibility and mission of school physical education have been put into the spotlight unconsciously, and school physical education has become the focus of whole society for a while. To reduce the pressure on all aspects of society, the school has to use the sword of examination-oriented education-examination and cancel the dangerous items in students' physical education class and the endurance items of long-distance running. However, teaching for the purpose of examination is obviously not the real education we pursue [9–11]. The development of the country depends on talents, and the cultivation of talents depends on education. In more than ten years since the implementation of quality education, our physical education is still full of difficulties. The measures taken by school physical education to avoid risks are undoubtedly inconsistent with people's all-round and healthy development.

School education is to integrate the precious spiritual wealth of mankind in history with today's social life and create a dynamic spirit and culture of the times [12, 13]. The study of physical education teaching evaluation is conducive to physical education workers making better decisions. Through extensive collection and analysis of information in teaching (information includes not only teachers, students, and other information in the education system but also information outside the education system, such as national guidelines, policies, and local culture) to determine the operation of next teaching practice activities [14]. Through teaching evaluation, teachers can first understand the problems existing in their teaching work, which is conducive to find out the weak links in the teaching process, to adjust the focus and direction of their next work, appropriately change teaching methods, formulate scientific teaching plans, better complete teaching tasks, and achieve teaching objectives; for students, evaluation is a kind of evaluation. Feedback correction system, through evaluation, can make students realize their progress and shortcomings in learning and help to clarify the priorities of learning in the next stage, to improve the effect of learning and guide students to achieve self-breakthrough [15–17]. For physical education, what kind of teaching evaluation methods and evaluation principles will have different effects on the development of students. Physical education is different from cultural courses. Physical skills and sports intelligence account for a large proportion of physical education, so it is necessary to evaluate physical education teaching and sports skills, but physical education also has the function of educating people, and it can also reveal many principles and philosophies of life, so the evaluation of humanistic thought and sports culture in physical education teaching is also indispensable [18–20]. This requires a more comprehensive, objective, and reasonable method to be applied to the evaluation of physical education teaching quality.

Physical education teaching and training involve many contents, so there are many comprehensive factors to be considered in its evaluation process, while the existing methods have the problem of low evaluation accuracy, and deep learning has the advantages of strong learning ability, wide coverage, strong adaptability, and good portability. Therefore, this study applies it to the quality evaluation of physical education teaching and training. The reliability of the evaluation results is verified by the application test. Through the research of this article, it is expected to provide a valuable reference for the evaluation of physical education teaching in colleges and universities.

## 2. Construct the Evaluation Index System of Physical Education Teaching

### 2.1. Current Situation of Physical Education Teaching Quality Evaluation

Whether it is the evaluation of physical education classroom teaching quality before the curriculum reform or after the curriculum reform, there are many places that need to be improved.

First, the evaluation criteria and indicators of physical education classroom teaching quality are only limited to the construction of theoretical framework and lack of empirical research on classroom behavior. This is a top-down theoretical derivation without the support of actual data from the front-line classroom. Such evaluation criteria and indicators lack accuracy in reliability and validity.

Second, the evaluation method is single, mainly qualitative evaluation, which lacks quantitative evaluation. It is more common to establish classroom teaching quality evaluation standards and index systems and then distribute the weights of the index systems, respectively. Such weight distribution is extremely subjective and lacks scientific distribution basis. It is only a theoretical classroom evaluation framework, not a substantive classroom evaluation tool, and cannot really reflect the accurate information and effect of classroom teaching. In addition, these evaluation index structures only express the commonness of classroom teaching but do not reflect the individuality of classroom teaching. Classroom teaching is a dynamic, generative, and open process with great personality differences. Therefore, you cannot measure all classes by the same standard. Moreover, in the actual classroom teaching evaluation, the evaluation standards and indicators are rarely used to evaluate the classroom teaching quality, and there is no research on the relationship between the evaluation standards and indicators and the classroom teaching effect. Over the years, although China's physical education classroom teaching quality evaluation has established many classroom teaching evaluation standards and indicator systems, it is only needed for the evaluation of open courses and high-quality courses. No teachers use these evaluation standards and index systems to guide actual teaching, and the promoting role of evaluation has not been brought into play.

Third is the lack of objective and accurate observation, recording, and description of physical education classroom teaching behavior. The new physical education curriculum emphasizes the feedback and incentive role of evaluation and the function of evaluation to promote students' development, teachers' improvement, and improvement of teaching. An accurate, objective, and comprehensive description of classroom teaching behavior is the prerequisite for teaching feedback and promoting the development of students and teachers. Only through the objective, accurate, and subtle observation and description of classroom teaching, we can make a profound analysis and judgment on classroom teaching, to evaluate, feedback, and improve classroom teaching; therefore, this study establishes a new method of PE classroom teaching quality evaluation based on deep learning.

### 2.2. Basic Principles of Constructing Physical Education Teaching Evaluation Index System

It can be seen from part 1.1 that the current physical education teaching evaluation system needs to be further refined and evaluated. Therefore, to further improve the quality of physical education teaching and training, the results given by the evaluation system are used, full play to its role in guiding actual teaching is given, and the physical education teaching evaluation index system is constructed.

Indicators are elements used to describe certain attributes of objective things. They are not only a specific and behavioral evaluation criterion but also a unit to measure objectives [21]. In the process of teaching quality evaluation, the evaluation index system is the basic basis for teaching evaluation, and whether the evaluation index system is scientific, reasonable, and direct affects the rationality and effectiveness of teaching quality evaluation results [22, 23]. This study realizes the construction of the evaluation system according to the following principles.

#### 2.2.1. Scientific Principle

The scientific principle means that in the process of teaching evaluation, the evaluation index system should have a certain theoretical basis, and each index should be consistent with the predetermined goal [24, 25]. Secondly, the concept description of each index should be scientific and accurate, the calculation scope should be clear, and the indexes closely related to teaching quality should be comprehensively analyzed and selected to make the index system as reasonable and effective as possible. The essential characteristics of the evaluation object are reflected.

#### 2.2.2. Feasibility Principle

The feasibility principle is that in the design process of teaching evaluation index system, the evaluation index should be simple and clear, the index content should have clear connotation and measurability, and the index data should be easy to obtain and simple to process, to ensure the smooth progress of the whole evaluation work.

#### 2.2.3. Principle of Comparability

The principle of comparability requires that the evaluation index must be the common attribute of all evaluation objects, reflecting the consistency of quality. In addition, since the quantity of different things can be compared with each other in quantity only after they are transformed into the same unit, the meaning, scope, and unit of measurement of the evaluation index must be consistent, to be comparable. The stronger its comparability, the better the final evaluation the more credible the result is.

#### 2.2.4. Accuracy Principle

The principle of accuracy means that when selecting evaluation indicators, we should follow the objective laws of teaching activities, combine the objective reality of teaching activities, and reflect the essence of teaching work. The selected evaluation indicators should have accurate connotation and extension and can accurately reflect the actual teaching situation of teachers. The established index system should be objective and credible and can accurately reflect the true of teaching evaluation.

#### 2.2.5. Principle of Independence

The principle of independence means that the indexes in the teaching quality evaluation index system should maintain a certain relative independence, not overlap or subordinate to each other. The indexes at the same level can only be parallel, and there can be no relationship between inclusion and inclusion or causality. The reason is that if the indexes in the index system are not independent of each other, then there will be redundant indicators, which will increase the workload of the whole evaluation process and reduce the feasibility of the evaluation results. In addition, if the indicators are included with each other, the indicators will be scored many times in the specific evaluation process, which will increase their corresponding weight and affect the final evaluation results.

### 2.3. Design of Evaluation Index System for Physical Education Teaching and Training Quality

According to the above five principles, the evaluation index system of physical education teaching and training quality is designed. This study uses the method of grey correlation analysis to realize this process. The basic idea of grey correlation is to select the reference sequence reflecting the index characteristics and the comparison sequence affecting the system behavior based on the mathematical basis of space theory and the four axioms of normalization, even symmetry, integrity, and proximity of grey correlation, then calculate the correlation coefficient and correlation degree between the dry comparison sequence and the ideal value, that is, the final reference sequence, and then sort and analyze the correlation degree, to get the corresponding results. Therefore, the index system is shown in Table 1.

Based on the evaluation index system designed in Table 1, it provides the basis for the follow-up evaluation of physical education teaching and training quality.

## 3. In-Depth Learning of Evaluation Indicators

To ensure that the specific evaluation results of the evaluation indicators have higher reliability, this study carries out in-depth learning, and the specific implementation steps are as follows.

### 3.1. Dimensionless Treatment of Original Indexes

In the evaluation index system of physical education teaching and training quality, due to the different orders of magnitude and dimension of each index factor, there is no comparability between each index data. Therefore, when carrying out grey correlation analysis, we should first deal with the original data dimensionless, eliminate the dimension, and then analyze and evaluate it. At present, there are many dimensionless processing methods, which can be summarized into three types: linear type, broken type, and curve type from the perspective of geometry. Considering the current situation of physical education teaching and training quality evaluation, this study adopts the linear dimensionless method to realize the process. Firstly, assuming that there are *m* factors affecting the final definition result of the index, and each factor has *n* data sequences, the original data sequence can be expressed as follows:

(1) $$\begin{array}{c} A={\left(a_{ij}\right)}_{m\times n}\left(\begin{array}{ccc} a_{11} & \ldots & a_{1n}\\ \vdots & \ddots & \vdots \\ a_{m1} & \cdots & a_{mn} \end{array}\right)\left(i=1,2,\ldots ,n;j=1,2,\ldots m\right), \end{array}$$

where *a*_*mn*_ represents the influencing factor of the evaluation index. The normalization of matrix *A* is realized by initial dimensionless, and its calculation formula is as follows:

(2) $$\begin{array}{c} a_{ij}^{\prime }=\frac{a_{ij}}{a_{1j}}. \end{array}$$

At this time, the corresponding dimensionless mean calculation formula is as follows:

(3) $$\begin{array}{c} \bar{a_{ij}^{\prime }}=\frac{a_{ij}}{\bar{a_{ij}}}. \end{array}$$

### 3.2. Setting of Resolution Coefficient

Based on the calculation results of dimensionless mean in part 2.1, in the calculation of correlation coefficient of index, the calculation method of resolution coefficient P can be expressed as follows:

(4) $$\begin{array}{c} p=\frac{\bar{a_{ij}}-\min\left(a_{ij}\right)}{\max\left(a_{ij}\right)-\min\left(a_{ij}\right)}. \end{array}$$

Among them, max(*a*_*ij*_) and min(*a*_*ij*_) represent the strongest and weakest factors affecting the evaluation index, respectively.

It can be seen from the above that the value of correlation coefficient mainly depends on the value of resolution coefficient *p*. Different values will inevitably lead to different values of final correlation coefficient, thus affecting the final ranking of correlation degree.

In combination with the above, if the resolution coefficient *p* is larger, the influence of each index factor on the correlation coefficient is greater, and the resolution of the corresponding correlation degree is smaller; if the value of *p* is small, the influence of each index factor on the correlation coefficient is small, and the resolution of the corresponding correlation degree is larger. Therefore, to ensure that the evaluation index system has better integrity and make the final correlation degree have better resolution, in the calculation process, the value of *p* is the maximum on the premise of stable data. When there are outliers in the observation sequence of each factor in the evaluation system, the correlation coefficient is almost completely dominated by max(*a*_*ij*_) and min(*a*_*ij*_). At this time, if the value of *p* is large, the values of correlation coefficients are very close to 1, so it is difficult to distinguish the similarity between the comparison data column and the reference data column, thus reducing the reliability of the final evaluation result. Therefore, in this case, in the calculation process, on the premise of stable data, the value of *p* is the minimum, to weaken the influence of max(*a*_*ij*_) and min(*a*_*ij*_). Then, it can be determined that the value range of resolution coefficient *p* is (0, 1].

The value range of the resolution coefficient *p* is obtained above. Therefore, this study designs the value principle of the resolution coefficient, mainly including the following three points. Firstly, its value makes the final correlation degree related not only to the absolute difference between the reference sequence and the comparison sequence but also to other index factors, to better reflect the integrity of the evaluation index system; secondly, when there are outliers in the observation sequence of each factor, the resolution coefficient can weaken its influence on the correlation coefficient, so that the correlation degree has the maximum amount of information and the maximum information resolution; finally, the resolution coefficient can be dynamically taken according to the change in the observation sequence, so that it has a certain objectivity and flexibility. On this basis, the calculation method of the resolution coefficient in this study is as follows:

(5) $$\begin{array}{c} p=\frac{1/n\sum_{i=1}^{n}\left|a_{ij}^{\prime }-\bar{a_{ij}}\right|}{\max\left(a_{ij}\right)-\min\left(a_{ij}\right)}. \end{array}$$

In this way, it is ensured that the final value result will not affect the integrity of the correlation degree of the final evaluation index system, and the resolution coefficient can be dynamically taken according to the actual situation, to provide a basis for the rationality of the final evaluation result.

### 3.3. Calculation of Evaluation Index Weight of Physical Education Teaching and Training

After determining the number of distinguished words of index factors, the association relationship *λ*_*ij*_ between indicators can be constructed. The association method in this study is as follows:

(6) $$\begin{array}{c} \sum \lambda_{ij}=\frac{1}{mn}\sum_{i=1}^{n}\sum_{j=1}^{m}p_{ij}, \end{array}$$

where *p*_*ij*_ represents the resolution coefficient between any two index influencing factors in the evaluation index system. It can be seen from formula (6) that the correlation degree is obtained by averaging the correlation coefficient. This means that the processing method ignores the weight of each index in the whole evaluation index system to a certain extent, which may lead to inaccurate final results. Therefore, in the calculation of correlation degree, this study uses the weighted method to take the sum of the product of the correlation coefficient of each index *λ*_*ij*_ and its corresponding weight *w*_*ij*_ as the quantitative expression of correlation degree, i.e.,

(7) $$\begin{array}{c} \tau =\lambda_{ij}w_{ij}=\frac{w_{ij}}{mn}\sum_{i=1}^{n}\sum_{j=1}^{m}p_{ij}. \end{array}$$

Among them, *w*_*ij*_ is the index weight determined according to the grey correlation analysis results of each index in the above. Using the systematic analysis method of grey correlation degree, which has described the size and strength relationship between various influencing factors, the steps to determine the weight of each evaluation index are as follows:

(1) Select Reference Sequence and Comparison Sequence. In the evaluation index system of physical education teaching and training quality, the selected reference standard is the optimal value of each evaluation index. In the selection process, according to the principle of “selecting the maximum value of benefit index and the minimum value of cost index,” the optimal value of each index is determined and used as the reference sequence, and the values of *M* secondary index factors in the teaching quality evaluation system are used as the comparison sequence.
(2) Determine the Weight of Secondary Index Factor. According to formula (7), the correlation coefficient and correlation degree are compared between the sequence and the reference sequence, the index weight at all levels is calculated, and the relative importance of each index is determined. Firstly, the hierarchical structure of the evaluation index system is constructed, with the primary index as the target layer, the secondary index as the criterion layer, and the tertiary index as the scheme layer. According to the relative importance level table, the evaluation indexes at the same level are compared, the support relationship between the upper and lower indexes is clarified, and the comparison judgment matrix is obtained. When the two indicators are equally important, the importance level is determined as 1; one indicator is slightly more important than the other, and the importance level is determined as 3; it is obviously more important than another index, and the importance level is determined as 5; it is more important than another indicator, and the importance level is determined as 7; it is extremely important than another indicator, and the importance level is determined as 9; between 1, 3, 5, 7, and 9, the importance level takes the intermediate value of adjacent judgment. The formula of judgment matrix *B* of indicators at the same level is as follows:
  (8) $$\begin{array}{c} B={\left(b_{ij}\right)}_{n\times n}, \end{array}$$

where *n* is the number of indicators compared in pairs at the same level and *b*_*ij*_ is the importance of the *i*th indicator relative to the *j*th indicator. Each column of *B* is normalized, the values of each column are summed, and then each value is divided by the sum of the columns. The resulting matrix *A*_*ij*_ is expressed as follows:

(9) $$\begin{array}{c} A_{ij}=\frac{b_{ij}}{\sum_{i=1}^{n}b_{ij}}. \end{array}$$

The value *a*_*ij*_ of each row of the original data sequence *A* obtains the matrix *D*_*i*_, the number of columns of the matrix is 1, and the expression formula is as follows:

(10) $$\begin{array}{c} D_{i}=\sum_{j=1}^{n}a_{ij}i,\;j=1\text{,}2\ldots n. \end{array}$$

The hierarchical weight *E*_*ij*_ of physical education teaching and training evaluation index is calculated, and the expression formula is as follows:

(11) $$\begin{array}{c} E_{ij}=\frac{m\times n}{\sum_{i=1}^{n}\sum_{j=1}^{m}w_{ij}}. \end{array}$$

Through formula (11), the single-level weight of physical education teaching and training evaluation index is obtained, and then, the index weight is calculated. The product of the index weight and the upper-level index weight is obtained to obtain the relative weight of each layer of evaluation index in the hierarchy for the overall goal of physical education teaching and training, that is, the weight value of the index relative to the overall goal. So far, the calculation of the weight of the evaluation index of physical education teaching and training has been completed.

## 4. Quality Model of Physical Education Teaching and Training

According to the above in-depth learning results, the evaluation index system is used to evaluate the quality of physical education teaching and training. In the specific evaluation process, there are two key points to pay attention to. The first is the distribution of students' sports performance and the growth range of students' sports performance. Based on this consideration, the evaluation function of physical education teaching and training constructed in this study is as follows:

(12) $$\begin{array}{c} F=\tau w_{ij}\left(\frac{\max c-\min c}{\max\;d-\min\;d}\right), \end{array}$$

where *F* represents the evaluation result, max*c*, min*c* represent the highest and lowest values of achievement, and max  *d*, min  *d* represent the maximum and minimum values of achievement progress.

In this way, the final evaluation result is obtained.

## 5. Empirical Analysis

### 5.1. Data Preparation and Processing

This study selects two classes of grade 2018 of a university as the test object and evaluates the physical education teaching based on the physical education test results of the two classes of students in the two semesters of 2018–2019. The items and time of the physical education test are the same. The two classes are recorded as A and B, respectively, with 30 students in each class. The specific teaching data are shown in Table 2.

Considering that the difference in sample data values is too large or too small, which will increase the computational complexity, it is normalized and scaled to the closed interval [0, 1]. On this basis, it is normalized, the weight of teaching quality evaluation index is determined, and the teaching purpose, teaching tools, and means are screened according to the weight value; based on the index data in Table 2, the following experiments are carried out through calculation and other processing.

### 5.2. Rating Results and Analysis

In order to ensure the effectiveness of the experiment, reference [7] based on ordered multiclassification logistic regression (MLR) model and reference [8] based on AHP-BPNN model are selected as comparison methods for the experiment.

**Figure 1:**
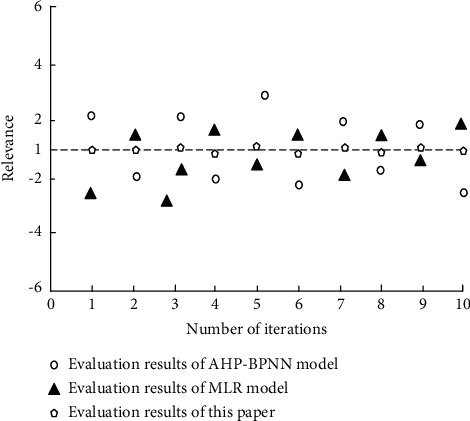
Correlation results of three effect evaluation methods.

According to the index correlation value calculated by formula (7), after repeated iterative calculation for 10 times, the calculated correlation value is sorted out, to verify the fitting degree of the three evaluation methods. Finally, the correlation between the participating evaluation indexes in the three evaluation methods is shown in Figure 1.

Corresponding to the constructed correlation value judgment results, when the defined correlation value is infinitely close to 1, it indicates that the indicators selected by this evaluation method are highly correlated. After repeated iterations for ten times, according to the data points calculated and processed in Figure 1, the correlation parameters calculated by the AHP-BPNN model are between −2 and 2, and the correlation of indicators involved in the evaluation process is poor. The correlation value calculated by the method in the MLR model is between −2.5 and 0.8, and the correlation of the selected indicators in the evaluation process is poor. The correlation index calculated in the iterative process of the designed evaluation method tends to be close to the value 1. Compared with the two existing evaluation methods, the correlation index selected in the evaluation process is the strongest.

**Figure 2:**
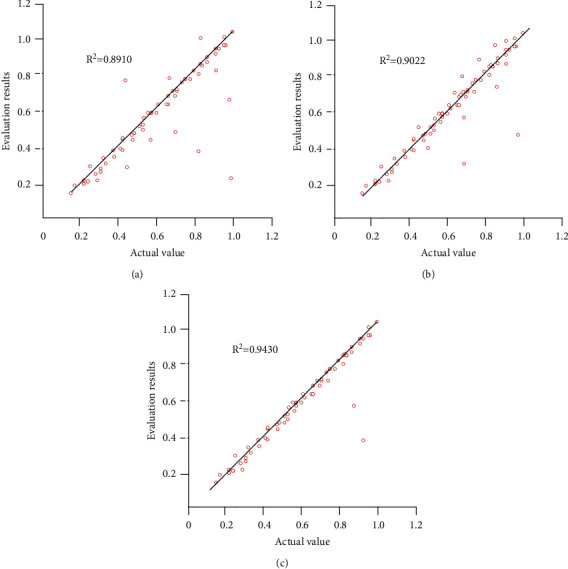
Comparison of evaluation results of different methods. (a) Evaluation results of the AHP-BPNN model. (b) Evaluation results of MLR model. (c) Evaluation results of this study.

The established optimal teaching quality evaluation method is used to detect the test set, and the AHP-BPNN model and MLR are used as the control group. At the same time, it is evaluated to obtain the actual output and model output, as shown in Figure 2.

According to the observation as shown in Figure 2, among the three methods, the correlation coefficient between the evaluation results and the actual result is 0.9430, the evaluation accuracy is 94.73%, and the evaluation results have high reliability. It shows that the teaching quality evaluation method based on deep learning designed in this study is effective and feasible. The deep learning algorithm covers all the 54 index data of physical education teaching evaluation, improves the coverage of evaluation indexes, and optimizes the evaluation performance.

To further compare the evaluation performance of the AHP-BPNN model, MLR model, and this method, the calculation results of resolution coefficient and correlation coefficient are compared, and the performance of each method is further verified by comparing the index values evaluated by each student. See Table 3 for the comparison results of performance index values evaluated by each method.

By analyzing Table 3, it can be concluded that the values of the resolution coefficient and correlation coefficient of the evaluation performance indicators of this method are the highest, and the average values of the two indicators are 0.9775 and 0.9742, respectively, while the average values of the two indicators of the AHP-BPNN model are 0.9442 and 0.9405, respectively, and the average values of the two indicators of MLR model are 0.9258 and 0.9198, respectively. Therefore, this method has better evaluation performance. The evaluation results are more accurate.

**Figure 3:**
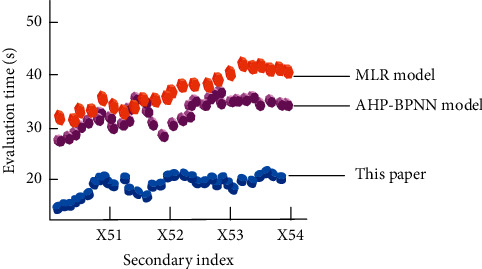
Comparison results of evaluation time of different methods.

The first-level index teaching effect is selected as the test object, and the evaluation time of different methods is tested, respectively. The results are shown in Figure 3.

By analyzing the experimental data in Figure 3, it can be seen that due to the different emphasis on secondary indicators included in the teaching effect of primary indicators, there are also obvious differences in the evaluation time of each method. Among them, the evaluation time of this method is the lowest among the three methods, followed by the AHP-BPNN model; the evaluation of the MLR model takes the most time. The experimental data fully show that the proposed method can complete the quality evaluation of physical education teaching and training at a faster speed, and the overall performance is obviously better than the other two evaluation methods.

## 6. Conclusion

Considering that the evaluation of physical education teaching quality will be affected and limited by many factors in the process of implementation, to carry out the evaluation of teaching quality smoothly and effectively, we must have a set of very reasonable evaluation index system, and the subject of evaluation must have the evaluation concept of advancing with the times. At the same time, physical education is the key link related to the healthy development of students' physique. Therefore, this study puts forward the evaluation method of physical education teaching and training quality based on in-depth learning and realizes the reliable evaluation of teaching quality through in-depth learning of evaluation indexes.

In the follow-up research, we can try to apply specific methods to further study the classroom teaching quality evaluation index system, to make it more scientific and rigorous.

## Figures and Tables

**Table 1:** Evaluation index system of physical education teaching and training quality.

Primary index		Secondary index	
Number	Name	Number	Name
X1	Teaching ability	X11	The explanation is clear and infectious
		X12	The theoretical concept description is accurate and the problem analysis is thorough
		X13	The curriculum design is reasonable and standardized
		X14	Understand the teaching content correctly and use it skillfully

X2	Teaching attitude	X21	Attend and finish classes on time, prepare lessons carefully, and be enthusiastic in class
		X22	Pay attention to and patiently coach and answer questions, carefully prepare lessons, and correct homework in time
		X23	Strict requirements for students in classroom discipline, homework, etc.
		X24	Timely understand the students' attendance and carefully listen to the students' opinions

X3	Teaching content	X31	Pay attention to integrating theory with practice, give appropriate examples, and update the teaching content on time
		X32	The teaching content is correct, substantial, deep, and broad
		X33	The teaching contents grasp the frontier of science and are related to scientific projects

X4	Teaching methods	X41	Pay attention to induction and summary and cultivate students' creative thinking
		X42	The course schedule is reasonable
		X43	Be able to use a variety of teaching methods to organize teaching (such as task-driven method and case teaching method)
		X44	Timely and effectively use modern educational means such as multimedia

X5	Teaching effectiveness	X51	Stimulate students' interest in learning and activate the classroom atmosphere
		X52	Students can understand and master the teaching content, basically achieve the teaching purpose, and complete the teaching task
		X53	Through teaching, students can enlighten students' thinking and improve students' ability to learn and solve problems
		X54	Students' physique has been improved

**Table 2:** Experimental data.

Number	X11	X12	X12	…	X54
1	96.23	75.69	86.24	…	68.77
2	95.24	86.61	88.92	…	69.52
3	86.24	92.37	73.69	…	73.45
…	…	…	…	…	…
60	88.42	86.31	72.19	…	73.60

**Table 3:** Comparison results of average values of evaluation performance indicators.

Algorithm	Number	Resolution coefficient	Correlation coefficient
Paper method	1	0.975	0.971
	2	0.973	0.972
	3	0.986	0.981
	4	0.982	0.978
	…	0.974	0.972
	60	0.975	0.971

AHP-BPNN model	1	0.943	0.938
	2	0.937	0.936
	3	0.948	0.945
	4	0.951	0.947
	…	0.944	0.941
	60	0.942	0.936

MLR model	1	0.927	0.922
	2	0.922	0.915
	3	0.923	0.917
	4	0.928	0.923
	…	0.934	0.928
	60	0.921	0.914

## Data Availability

Some or all data, models, or code generated or used during the study are available from the corresponding author by request.
